# How HIV Takes Advantage of the Cytoskeleton in Entry and Replication

**DOI:** 10.3390/v3040293

**Published:** 2011-03-28

**Authors:** Bettina Stolp, Oliver T. Fackler

**Affiliations:** Department of Infectious Diseases, Virology, University Hospital Heidelberg, Im Neuenheimer Feld 324, D-69120 Heidelberg, Germany; E-Mail: bettina.stolp@med.uni-heidelberg.de

**Keywords:** HIV, actin cytoskeleton, entry, Nef, cofilin

## Abstract

The host cell cytoskeleton plays a key role in the life cycle of viral pathogens whose propagation depends on mandatory intracellular steps. Accordingly, also the human immunodeficiency virus type 1 (HIV-1) has evolved strategies to exploit and modulate in particular the actin cytoskeleton for its purposes. This review will recapitulate recent findings on how HIV-1 hijacks the cytoskeleton to facilitate entry into, transport within and egress from host cells as well as to commandeer communication of infected with uninfected bystander cells.

## 1. Introduction

Dynamic cellular processes such as cell motility, cell division, intracellular transport and endocytosis depend on the cytoskeleton comprised of actin, microtubule and intermediate filament systems [1–3]. As obligate intracellular pathogens, viruses rely on host cell machinery for most steps in their replication cycle and thus depend on functional interactions with cytoskeletal elements. Consequently, viruses have evolved optimized replication strategies that include manipulations of the cellular actin and microtubule cytoskeleton in their host cells. In the case of the human immunodeficiency virus type 1 (HIV-1) the actin cytoskeleton represents a significant physical barrier during virus entry. Furthermore, intracellular transport of incoming genomes towards the nucleus and of virion components to budding sites appears to require functional interactions with the host cell cytoskeleton. Finally, cytoskeletal manipulations are employed as a convenient tool to tailor communication of infected cells with the environment. To this end, the HIV-1 encoded structural proteins Gag and Env but also the accessory proteins Nef, Rev, Tat and Vif have been reported to interact with and/or affect host cell cytoskeletal structures. In this review we will summarize our current understanding of HIV-1’s interaction with the host cell cytoskeleton with a particular emphasis on events during virus entry as well as on the role of the Nef protein as an inhibitor of F-actin remodeling.

## 2. The Role of the Cytoskeleton in the HIV Life Cycle

### 2.1. Entry

**Figure 1.**
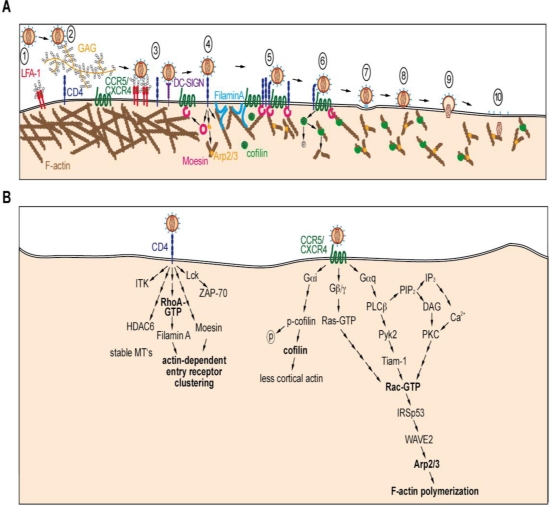
Proposed signaling pathway to the actin cytoskeleton during HIV-1 entry in T lymphocytes. (**a**) Mature free virions (step 1) are initially captured by unspecific binding to glycoproteins covering the target cell such as glycosaminoglycans (GAG, step 2) or cell surface molecules such as LFA-1 or DC-SIGN (step 3). Subsequent specific binding of gp120 to CD4 initiates signaling to moesin, Arp2/3 and filaminA (step 4), resulting in entry receptor clustering (step 5). This allows gp120 to engage chemokine entry coreceptors to initiate receptor signaling, whose downstream effects include activation of cofilin (step 6). Active cofilin and Arp2/3 locally loosen the cortical actin cytoskeleton and provide motility (steps 7 and 8), to facilitate release of the viral core into the cytoplasm and transport through the cortical actin, respectively (steps 9 and 10). (**b**) Schematic representation of the current understanding of signaling cascades induced by gp120 through binding to CD4 (left panel) and CXCR4/CCR5 (right panel) during HIV-1 entry. Note that these pathways may be distinct in HIV-1 target cells such as macrophages that lack expression of T cell specific factors such as Lck.

Entry of HIV-1 into target cells is a highly coordinated multi-step process that is understood in great molecular detail [4,5]. Initial cell attachment of HIV-1 mainly occurs via unspecific binding to lectins, glycosaminoglycans or via receptor-ligand interactions of virion incorporated cell surface proteins [6–8] (Figure 1A, steps 2 and 3). Subsequent entry requires binding of the viral envelope glycoprotein gp120 to its primary entry receptor CD4, resulting in a conformational change in gp120 that exposes the binding site for one of the HIV-1 entry co-receptors CCR5 or CXCR4. Co-receptor binding induces gp41 fusion peptide insertion into the host cell membrane and subsequent six-helix bundle formation approaches viral and cellular membranes, resulting in membrane fusion and subsequent release of the viral core into the cytosol (reviewed in [9]). The cortical actin cytoskeleton is involved in this process at least in two steps: First, during the initial phase in which entry receptors need to be clustered for efficient entry (Figure 1A, step 5). Second, following the fusion reaction, cytoskeletal rearrangements facilitate release of the viral core into the cytoplasm (Figure 1A, steps 9 and 10). In both cases, HIV-1 modulates these events by binding of gp120 to its receptor and coreceptor, which elicits signaling pathways that alter dynamic cytoskeleton remodeling (Figure 1). The exact spatiotemporal sequence of these signaling events is poorly understood, but it is well accepted that HIV binding induces signaling cascades similar to the natural receptor ligands. We will summarize below our current understanding of these cytoskeletal-dependent steps in HIV-1 entry.

#### 2.1.1. Entry Receptor Clustering and Cytoplasmic Delivery of Virion Cores

One of the first challenges incoming HIV-1 virions meet on their way to productive infection is to locally enrich CD4 and co-receptor for coordinated membrane fusion (Figure 1A, step 5). Several studies indicate that HIV-1 solves this problem by inducing a signaling cascade that triggers actin-dependent clustering of HIV-1 entry receptors to reduce their lateral mobility and thus to establish signaling platforms that stabilize fusion pore formation [10–13]. This is achieved by binding of gp120 to CD4 and the chemokine entry co-receptors which induces recruitment of the membrane-actin crosslinker filaminA to physically link the cytoplasmic tails of these receptors to F-actin [10,13]. This stabilization facilitates subsequent conformational changes of gp120 required for membrane fusion. Importantly, gp120-receptor interactions *per se* do not automatically result in reduced receptor mobility. This fact is exploited by retroviruses including HIV-1, which, while bound to their entry receptors at the target cell surface, benefit from actin-driven mobility of these receptors to reach subcellular sites with optimized conditions for virus entry [14]. How this process that is referred to as “virus surfing” is coordinated with arrest of receptor movement and triggering of fusion will be an interesting area of research in coming years.

Subsequent to successful clustering of entry receptors and initiation of membrane fusion, the cortical actin cytoskeleton represents a barrier to the delivery of viral cores into the cytoplasm. This hurdle is again overcome by signals derived from gp120-receptor interactions, in particular with the chemokine co-receptors. Chemokine receptors are a large family of seven transmembrane serpentine receptors that are coupled to heterotrimeric G proteins on the inner leaflet of the plasma membrane to trigger signaling upon engagement. Downstream effector cascades are predominantly determined by the nature of the coupled heterotrimeric G proteins. In the case of HIV-1 gp120 and CXCR4/CCR5, signaling seems to occur mainly via Gα_q_ or Gα_I_ [15,16]. Physiological chemokine receptor-Gα signaling typically results in the activation of Rho-GTPases via induction of a complex signaling pathway (Figure 1B, right panel). HIV-1 mimics this cascade and triggers Rho GTPase activation, however which specific GTPases are targeted is still a matter of debate. Roles for activation of RhoA as well as Rac1 have been reported [13,15,17–20]. These findings are not mutually exclusive and may reflect that RhoA activation occurs via CD4 engagement, whereas Rac1 is activated downstream of chemokine co-receptors. Irrespective of the specific GTPase involved, many components of the signaling pathway have been identified (Figure 1B) that directly target the actin remodeling machinery of the host cell. One important target appears to be the Arp2/3 complex, a large protein assembly regulated by Rac1 that exerts nucleation activity for the *de novo* formation of F-actin filaments. Arp2/3 activity is induced by HIV-1 Env-chemokine receptor interactions, an activity essential for efficient HIV entry [18,21]. More recently, the actin severing factor cofilin has been identified as an additional target of Env-mediated signaling during HIV entry and its activity is regulated in a biphasic manner. Cofilin is phosphorylated and thereby inactivated via a RhoA and Rac-dependent pathway downstream of CD4 and CXCR4, respectively, within seconds of HIV binding to a target cell [13,22]. Since cofilin inactivation reduces actin remodeling, this effect may contribute to efficient receptor clustering. In subsequent steps of HIV-1 entry, cofilin is dephosphorylated and thus activated, leading to the fragmentation of existing actin filaments and thus presumably loosening of the actin cortex [16,23]. This rapid switch of cofilin activation states during HIV-1 entry resembles those observed following physiological chemokine receptor engagement where a rapid (within seconds) and transient phosphorylation is followed by a prolonged (20–30 minutes) phase of cofilin activation [22,24–26]. This elegant combination of activation of Arp2/3 and cofilin conceivably results in enhanced actin remodeling in the cell periphery, which is thought to facilitate delivery of the viral core through the otherwise rigid cell periphery. Movement of viral cores near the plasma membrane has been shown to be actin and microtubule dependent [27,28] and may be initiated by cofilin-dependent free barbed end production and Arp2/3 facilitated F-actin polymerization.

Even though signaling pathways elicited by CD4 and the co-receptors are not easy to distinguish due to overlapping downstream signaling pathways, evidence based on the use of receptor specific blocking antibodies is emerging that CD4 binding also triggers selective signaling that contributes to efficient HIV-1 entry. At early entry steps, CD4 engagement has been shown to activate the membrane-actin crosslinking protein moesin to facilitate entry receptor clustering and thus efficient entry [12] (Figure 1A, steps 4–6). Similarly at later steps, CD4 specific signals trigger, via a yet to be resolved mechanism, the inactivation of the histone deacetylase 6 (HDAC6), which results in an increased level of acetylated and thus stabilized microtubules (Figure 1B, left panel). Possibly by stabilizing fusion pores for delivery of viral cores to the cytoplasm, formation of these rigid structures potently facilitates productive HIV-1 entry [29].

#### 2.1.2. Sensitization of Uninfected Bystander Cells for Entry

Consistent with the analogy between signaling triggered by HIV-1 co-receptor chemokine ligands and gp120, pretreatment of HIV permissive cells with chemokines binding to the HIV co-receptors facilitates replication by priming the cell for subsequent infection [23]. Recent results indicate that HIV-1 exploits this phenomenon to prime uninfected non-activated CD4^+^ T lymphocytes in infected patients, a cell population that is typically rather refractory to viral infection. This is achieved by shedding of gp120 molecules from productively infected cells, resulting in elevated serum concentrations of gp120 sufficient for triggering of baseline chemokine receptor signaling. Similar to events during the actual infection process, this may enhance cofilin activity to increase the turnover of cortical actin and thus to facilitate passage of incoming virions through the otherwise rigid actin cortex [23,30]. It thus emerges that HIV-1 may have specifically evolved loose interactions between its glycoprotein subunits that lead to shedding of gp120, thereby recycling the mechanism involved in productive entry of immediate target cells for priming of future targets.

#### 2.1.3. Requirement for Cytoskeletal Interactions during Endocytic Entry?

Historically, HIV-1 entry was believed to occur predominantly or even exclusively at the plasma membrane [5]. More recently, however, evidence accumulates for a prominent role of fusion with endosomal membranes following endocytosis in productive HIV-1 infection [4,20,31–36]. Most studies on the role of the host cell cytoskeleton in HIV-1 entry did not distinguish between productive entry events occurring at the plasma membrane or following endocytic uptake. It remains thus unclear whether endocytic and plasma membrane entry routes of HIV-1 differ in their requirements for actin remodeling. The endocytic route arguably provides incoming virions with the advantage that, as cargo of an endocytic vesicle, the cortical actin barrier can be overcome without a need for virus-induced cytoskeletal rearrangements. While the actin cytoskeleton does not seem to play an essential role for the budding of such endocytic vesicles, evidence is accumulating that specific actin nucleation machineries are recruited to individual types of endosomal vesicles to drive their motility and thus possibly productive HIV-1 infection [37,38]. However, the precise endocytic route via which HIV enters target cells is only poorly defined and endocytic pathways rely to different extents on dynamic actin remodeling for uptake and intracellular trafficking [39]. Moreover, endocytic entry of HIV-1 likely occurs via very distinct uptake routes in diverse target cells such as T lymphocytes and macrophages and the contribution of these pathways for productive infection will likely be distinct [4,20,32,33]. With most studies on endocytic HIV entry focusing on adherent cells with engineered overexpression of the HIV-1 entry receptor complex, the delineation of the relevance of individual entry pathways in physiological target cells including primary human T lymphocytes will be an important future goal. The involvement of specific cytoskeletal elements will serve as a valuable tool in these studies that may allow discrimination between individual routes employed. Finally, it is becoming increasingly clear that HIV-1 predominately spreads via cell-cell contacts rather than by infection of cell-free virus particles. HIV-1 cell-cell transmission clearly depends on the integrity of the microtubule network and is mediated by F-actin rich, dynamic cell protrusions that may also be subject to endocytosis in target cells during productive entry [4,40–43].

### 2.2. Transport to Nucleus

Delivery of the viral core into the cytoplasm is followed by uncoating of the viral genome to release it into the host cell cytoplasm where it resides in association with viral proteins in a structure referred to as the preintegration complex (PIC). This step has remained the most enigmatic in the life cycle of HIV-1 and the molecular mechanism and the subcellular site of uncoating, as well as the composition of the PIC are debated [44–46]. Irrespective of the detailed uncoating mechanism, movement of the PIC by brownian motion or diffusion is restricted by the densely packed cytoplasm [47] and translocation to the nucleus therefore requires active transport processes, the nature of which is only slowly beginning to emerge. Both actin and microtubule dependent transport events have been proposed to play a role [27,28,48]. In this scenario, initial transport through the cortical actin at the cell periphery is thought to rely on actin-based motility possibly mediated by the Arp2/3 complex, subsequently followed by rapid microtubule dependent delivery towards the nucleus. However, this model is mostly based on the imaging of individual incoming viral components in association with cytoskeletal elements and how relevant these individual particles are to productive infection is difficult to assess. The identification of specific host cell and viral components that govern these individual transport steps will greatly contribute to our understanding of this important step in the HIV-1 life cycle.

### 2.3. Transcription and Nuclear Export

Even though individual viral genes are already expressed to some extent from incoming cytoplasmic genome copies [49], transcription of the full set of viral genes is only initiated following nuclear import and integration of the viral genome into the host cell chromosome. HIV-1 transcription is an orchestrated process that depends on the viral transactivator Tat together with several host cell transcription initiation, elongation and termination complexes [50]. Little is known about the potential involvement of the host cell cytoskeleton in this process. However, cytoplasmic remodeling of e.g., actin filaments is known to affect the availability of cytoplasmic transcription factors such as MAL for translocation into the nucleus and thus for transcription of specific target genes [51]. Moreover it is becoming increasingly clear that actin is an abundant component of cell nuclei, where it is subject to complex dynamic regulation that may directly impact on transcriptional activity [52,53]. Such effects could be directly affected by Tat, which has been reported to affect polymerization states of F-actin [54,55]. The promoter of the integrated HIV-1 genome is partially condensed into nucleosomes to prevent efficient transcript elongation and it is well established that Tat recruits histone acetyltransferases (HATs) such as p300/CBP that locally loosen chromatin during transcription [56]. Moreover, ATP-driven chromatin remodeling complexes of the SWI/SNF family are also recruited to the HIV-1 promoter region, where they cooperate with HATs to allow efficient HIV transcription [57]. Importantly, β-actin and actin-related proteins represent crucial components of those complexes and co-immunoprecipitate with HIV-1 Tat, indicating that actin and actin binding molecules may constitute crucial components of the HIV transcriptional machinery [57,58]. Defined signaling mechanisms, the role of cytoplasmic as well as nuclear actin turnover and the precise set of actin binding proteins involved in this process, however, remain to be unraveled. Since the nuclear export factor for unspliced viral RNA, Rev, depends on and colocalizes with nuclear actin-bundles to allow efficient viral export, nuclear actin may also play pivotal roles during Rev-dependent nuclear export of genomic viral RNA [59]. The advent of novel experimental tools that allow the specific visualization and manipulation of nuclear actin [52,53] opens avenues for fascinating studies on the involvement of such mechanisms in the HIV-1 life cycle.

### 2.4. Budding/Assembly/Release

Similar to the events during entry of HIV-1 particles in new target cells it could be assumed that cytoskeletal structures mediate transport of individual virion components to budding sites at the plasma membrane of producer cells and that the cortical actin meshwork constitutes a substantial barrier to the release of newly formed virions. However, all available evidence indicates that the late steps in the HIV replication cycle depend remarkably little on the host cell cytoskeleton. In particular, treatment of HIV-1 producing cells with actin and microtubule disrupting drugs cause little inhibition of HIV-1 production [60,61]. Moreover, based on live cell imaging of virus producing HeLa cells, addition of the actin disrupting drug LatrunculinA does not exert apparent effects on the position of HIV-1 budding structures or the assembly kinetics at individual budding sites [62]. These findings raise the question how Gag and the viral genomic RNA are trafficked through the cytoplasm to the plasma membrane prior to budding. While this is currently unclear for the HIV-1 genomic RNA, Gag could reach the plasma membrane by free diffusion in its monomeric form followed by PI(4,5)P_2_ dependent recruitment to the plasma membrane where assembly occurs [63,64]. However, associations of Gag with actin [65–67] as well as with the microtubule motor KIF4 [64,68] and of the RNA genome with proteins that provide a link to the cytoskeleton [69] have been reported, the functional relevance of which remain to be delineated. While the cytoskeleton may thus be directly or indirectly involved in the transport of virion components, the actual budding process does not appear to strictly rely on cytoskeleton remodeling. In this scenario, the observed colocalization of F-actin with budding structures [70] and the packaging of actin and actin binding proteins into HIV-1 virions [71,72] would be secondary consequences of the high abundance of these molecules at budding sites. This raises the question of how budding HIV-1 virions circumvent the physical block imposed by the actin cortex. In principle, monomeric Gag proteins could simply diffuse through the cortical actin and assemble underneath the plasma membrane, thereby possibly bypassing the diffusion restriction imposed to larger, pre-assembled complexes. In addition, hijacking of the cellular ESCRT machinery that is essential for late assembly steps and subsequent release of HIV [73] may provide HIV-1 with a built-in mechanism that locally destabilizes F-actin for efficient release. Indeed, remodeling of the actin cortex is observed at HIV-1 budding sites [70] but may, based on the lack of potent effect of actin remodeling inhibitors, not be essential for particle release. The role of the cytoskeleton in ESCRT function, e.g., during the final steps of cytokinesis [74], has so far not been exhaustively analyzed, but initial findings in *Drosophila* indicate that components of the ESCRT machinery are able to interact with the actin cytoskeleton [75].

## 3. HIV-1 Accessory Proteins and Their Influence on the Cytoskeleton:

The accessory genes of HIV-1 have regulatory functions and facilitate viral replication by optimizing the environment for the virus in the infected host. For the HIV-1 accessory proteins Vpu and Vpr, no relevant functional interactions with the host cell cytoskeleton have been reported thus far. The viral infectivity factor Vif can associate with intermediate filaments [76], whether this is relevant for its role as antagonist of the host cell restriction factors of the APOBEC protein family however remains to be addressed [77]. In contrast and as discussed in the following in detail, the HIV-1 accessory protein Nef emerges as important regulator of the cellular actin cytoskeleton in infected cells.

### 3.1. Nef

#### 3.1.1 Functions of Nef in HIV-1 Infection

The lentiviral Nef protein is an important pathogenicity factor that elevates virus replication *in vivo* [78]. Numerous independent functions have been attributed to this protein *in vitro*, including alterations of intracellular trafficking, modification of cellular signaling and enhancement of virion infectivity [79–81]. How these individual activities synergize to promote virus spread and pathogenesis *in vivo* remains to be fully resolved. Effects of Nef on F-actin structures were first observed in murine fibroblasts where Nef, when expressed with the co-factor Vav, induced potent actin depolymerization [82]. Subsequently similar effects of Nef on F-actin structures were reported for HIV-1 target cells overexpressing Nef or following HIV-1 infection, and even upon extracellular addition of recombinant Nef protein. Genetic approaches revealed that the effects of Nef on actin remodeling are not essential for many other Nef activities, thus establishing the interplay of Nef with F-actin as an independent function of the pathogenicity factor [83–86].

#### 3.1.2. Mechanism of Actin Remodeling Inhibition by Nef

**Figure 2.**
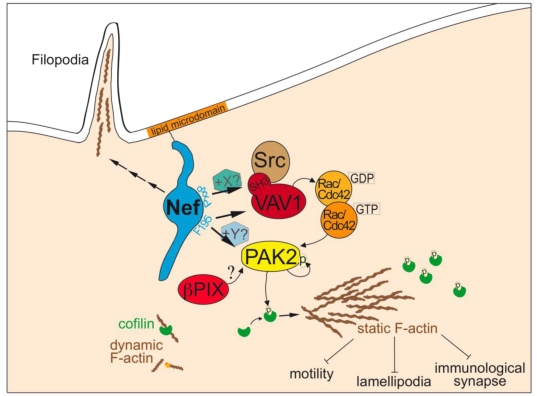
Nef prevents F-actin turnover in T lymphocytes. Nef is incorporated into lipid microdomains via its myristoyl anchor and associates with a labile multiprotein complex of about 1 MDa, which contains the cellular kinase PAK2, VAV1, a small Rho GTPase and several unknown factors. Interaction surfaces required for complex assembly are known, the precise interaction partners, however, are unclear and association may be mediated by yet-to-be identified factors (X, Y). Nef associates with a highly active subpopulation of PAK2 and redirects it towards the phosphorylation of cofilin to block actin dynamics. This inhibition appears to be specific for individual F-actin rich protrusions such as membrane ruffles/lamellipodia while the formation of filopodia is not affected by Nef.

Mapping and functional experiments established that modulation of F-actin organization by Nef requires its association with cellular membranes as well as with the cellular kinase PAK2 [82,83,85,87]. Over the past years, significant progress was made towards understanding how Nef exploits PAK2 to modulate host cell actin dynamics. The Nef-PAK2 association occurs in the context of a fragile multiprotein complex that contains a highly active subpopulation of PAK2, the Rho-GTPase Rac1, its guanine nucleotide exchange factor Vav1, as well as several unknown proteins [82,88]. Assembling Nef-PAK2 complexes in lipid raft membrane microdomains [89,90], Nef employs its SH3 domain binding motif as well as a patch surrounding a critical phenylalanine near its C-terminus for the recruitment of host cell components [86,91–93]. Even though the identity of the direct interaction partners of these motifs remains to be established, mutation of the critical phenyalanine resulted in a mutant Nef protein that is selectively deficient for association with PAK2 and thus for interference with host cell actin dynamics, but maintains wild type activity for all other Nef functions [86,91]. Use of this mutant along with silencing of PAK2 demonstrated that association with PAK2 is crucial for Nef’s inhibitory effects on the actin cytoskeleton [83,85,87]. A recent mechanistic study showed that Nef alters the substrate specificity of PAK2 towards the actin severing factor cofilin. Not a PAK2 substrate in the absence of Nef, phosphorylation of cofilin causes its inactivation in HIV-1 infected cells, resulting in reduced actin turnover [83,87]. This Nef function is highly conserved among different *nef* alleles of HIV-1, HIV-2 and SIV [87, 94] and can be observed in a wide variety of cell types of different species origin [82,83,95,96]. Interestingly, Nef does not affect basal F-actin levels or morphology of quiescent cells via this mechanism but primes cells to be non-responsive to most external triggers that induce actin remodeling. Moreover, inhibitory effects of Nef appear restricted to sheet-like actin protrusions as observed in chemokine-induced plasma membrane ruffles [83,87,97], circumferential F-actin rich rings [84,85] and at the immunological synapse (IS) [94] (Figure 2). In contrast, Nef does not exert such inhibition on thin F-actin rich protrusions such as filopodia or nanotubes observed during cell-to-cell transmission of HIV [97,98] (Figure 2).

#### 3.1.3. Functional Consequences of Altered Actin Dynamics in T Lymphocytes

In T lymphocytes, actin remodeling upon T cell receptor (TCR) engagement or chemokine stimulation was identified to be subject to Nef-mediated inhibition [83–85,87,94]. The ability of T cells to intimately interact with antigen presenting cells (APCs), by forming a TCR signaling induced IS, is central to the initiation of an immune response [99]. One hallmark of this interaction is the pronounced F-actin accumulation at cell-cell contacts. In the case of ISs formed between B cells and cognate T cells, this contact is critical for TCR signaling and thus T cell activation. Nef proteins from HIV-1, HIV-2 and SIV potently interfere with F-actin remodeling at the IS between infected T cells and superantigen-loaded B cells [94]. In addition, Nef also affects intracellular transport events required for efficient TCR signaling, thus resulting in a marked reduction in TCR proximal signaling events [84,100,101]. Despite these blocks to the early steps in the TCR cascade, distal TCR signaling is remarkably efficient in HIV-1 infected T cells, while Nef proteins from HIV-2 and SIV efficiently shut down signaling by reducing cell surface exposure of CD3-TCR complexes [102]. Via this mechanism, HIV-1 Nef may ensure elevated levels of T cell activation required for efficient virus replication while preventing T cell hyperactivation and concomitant activation-induced cell death that would limit virus spread [103]. Addressing how HIV-1 Nef maintains distal TCR signaling while disrupting initial events at the IS and which precise role inhibition of actin remodeling plays in this context represent important questions for future studies.

Besides interfering with TCR-induced actin remodeling, Nef also affects chemokine induced actin remodeling and accompanied chemotaxis [83,87]. Reflecting the strict requirement of cell motility for a precise spatiotemporal control of actin dynamics [3,104], Nef potently interferes with cell motility in response to various stimuli in different cell types [83,87,97,105]. Dynamic actin turnover and efficient motility are also central to T lymphocyte immune functions *in vivo* such as screening for antigen, homing to lymph nodes as well as activation and interaction with other immune cells [106]. We therefore propose that Nef, via inhibition of T lymphocyte chemotaxis, prevents HIV-1 infected T lymphocytes from homing into lymph nodes and scanning for cognate antigen. Such failure in migration towards the T cell area in the lymph node will prevent these T lymphocytes from encountering B cells, which is crucial B cell activation and subsequent formation of germinal centers, where immunoglobulin class switching and production of high affinity antibodies occurs [107]. Consistent with this model, SIV wildtype infected cells are retained in the paracortex of lymph nodes of SIVmac239-infected macaques and germinal center formation is prevented, whereas SIVΔNef infected cells readily migrate into the area of the germinal center [108]. Nef-mediated inhibition of actin remodeling and cell migration may thus interfere with the production of high affinity antibodies, thereby contributing to the B cell dysfunction frequently observed in AIDS patients [109]. Since not only movement within but also egress from lymph nodes back into the blood circulation represents an active process that depends on the migratory capacity of cells, Nef-mediated inhibition of T lymphocyte motility may also retain infected cells inside lymph nodes, thereby keeping them in near proximity to susceptible target cells [110]. HIV-1 spread in densely packed lymphoid organs is thought to occur predominantly via the cell-to-cell route [111,112], a process that relies on F-actin rich cytoneme bridges and is per se not affected by Nef [40,98,113,114]. Reduced motility of infected cells may however indirectly favor virus transmission to new target cells and account for enhanced virus replication in the presence of Nef *in vivo*. Together, inhibition of triggered actin remodeling by Nef emerges as versatile and important strategy of HIV-1 to adjust T lymphocyte homeostasis in order to facilitate viral spread and to impair the immune system of the host, thereby creating an optimal niche for viral replication.

#### 3.1.4. Nef-Mediated Alteration of F-Actin Organization in Other Cell Types

Besides T lymphocytes Nef also alters actin organization in other HIV-1 target cells by yet-to-be established molecular mechanisms. In macrophages, filopodia like structures serve as long-range conduits to transport Nef over long distances inside the lymph node to transfer it to B cells that do not represent HIV-1 target cells [115]. Whether Nef itself induces filopodia on macrophages or whether existing structures are hijacked is currently unclear. B cells have been shown to contain Nef in lymph nodes of HIV-infected patients and Nef interferes with immunoglobulin class switching, thereby interfering with the production of high affinity antibodies [115,116]. This effect may synergize with the Nef-mediated inhibition of T lymphocyte chemotaxis and effects on B cell activation via soluble factors [117], suggesting that manipulation of B cell function may constitute a cardinal function of Nef in AIDS pathogenesis. Finally, effects of Nef on actin organization have also been described for podocytes and dendritic cells (DC). Podocytes are specialized cells in the kidney epithelium that can be infected by HIV-1 and exhibit an altered actin cytoskeleton upon expression of Nef [118]. The observed phenotype however clearly differs from that observed in T lymphocytes as lamellipodia and F-actin stress fibers are induced and may involve a distinct molecular mechanism that relies on the interaction of Nef with the diaphanous interacting protein. Finally, induction of F-actin rich plasma membrane ruffles and uropods have been observed following incubation of DCs with extracellular Nef [119], however mechanism and functional relevance of this phenomenon have not yet been explored.

## 4. Conclusion

HIV-1 takes advantage of the cellular cytoskeleton at various steps in its replication cycle, most intriguingly is the involvement of the actin machinery during the entry process. However, knowledge is limited at other steps of the replication cycle and future work will be required to shed light on these black boxes of HIV replication. The accessory protein Nef emerges as a major regulator of the actin cytoskeleton in an HIV infected cell and we are only beginning to understand how the virus benefits from hijacking host cell actin dynamics. Improved imaging techniques and labeling strategies as well as the development of new experimental tools will allow us to further dissect the role of the cellular cytoskeleton for HIV-1 replication and pathogenesis.
